# Boston Letter

**Published:** 1880-11

**Authors:** 


### Domestic Correspondence.

### ARTICLE IX.

### BOSTON LETTER.

*Messrs. Editors* :—In looking over our city directory for 1880, I was surprised to find that three hundred and eighty-two members of the Massachusetts Medical Society practice medicine in the City of Boston alone. This of course includes the Charlestown, Roxbury and Dorchester districts, which are now a portion of the city. But since the Fellows of our State Society number only twelve or thirteen hundred, it will be seen that a large proportion of them are located within a comparatively small section of the whole State. It cannot be supposed that all of these are blessed with a remunerative practice. The directory contains the names of seventy Homœopaths, so called, six of whom are women. There are also fourteen Eclectics ; six names under the auspices of the United States Medical Society of Massachusetts ; twenty-seven members of the "New England Medical Society of Specialists ;" twelve members of the New England Hospital Society, all of whom are women who, I believe, are regular in their practice ; one hundred and fifteen female practioners who are not included under any organization, and who practice all sorts of 'isms and 'pathies, from hair-dressing to midwifery, and finally I find a list of doctors headed "Other Physicians" of whom there seem to be two hundred and fifty-four. "Other physicians" is an awkward squad, to be a member of which is not a specially high honor. Some of these left-handed lights of medicine indicate their curious forms of practice by names which are as puzzling as Barnum's "What is it ?" but partake far more of the *genus* humbug. One fellow's practice is "analytical,"

another's "anatomical," that of a third "naturepathic," of a fourth "psycopathic." There are also "bonesetters," "magnetics," "electrics," "botanics," many of whom no doubt flourish upon that singular credulity which from time unknown has been a characteristic of the common people. Several of these men are Buchananites, their names having been published in the list of the graduates of the Buchanan mills at Philadelphia. It is a question whether, in the absence of State medical laws, these scamps should be posted in their several localities. This would merely advertise them without accomplishing anything in behalf of their victims. Among "other physicians" too, I am sorry to say, are men who are advertising abortionists, and belong to that class who make pitiable dupes of the weak and erring of both sexes. Why they are allowed to pursue their nefarious trade and advertise beside, may perhaps be explained by the frank confession of a man who quietly follows gambling as a business. He said to an acquaintance of mine that so long as he paid a certain yearly amount to the police of his district, he was not troubled, and that he considered the police tax a legitimate (?) item in his expenses. It is more than probable that abortionists protect themselves in a similar manner. At any rate it is a well-known difficulty to find proof against them. The mortifying fact is made evident by this classification, that in Boston out of nearly nine hundred individuals who are called doctors, less than four hundred are regular. If we ever push through the legislature of Massachusetts a law regulating the practice of medicine it will be doubtful as to how successful it can be made. At any rate I suppose no law can prevent the employment of quacks by the laity if the latter prefer quacks to educated men.

Just as business houses of a similar kind cluster together in the same street, so in Boston do physicians live in groups. On two of our principal streets if a person were to call out "doctor" at midnight, the result would probably exceed that accomplished by the cross-eyed schoolmaster, who said, "The boy I am looking at will please stand." Twenty-nine boys rose to their feet. For, in one of these streets, within one and a half blocks, live about sixty physicians.

At a recent meeting of the councilors of our State Society, the proposition was made that the president should be requested to appoint a committee to consider the necessity of a law for the protection of the insane during the interval between the signing of the certificate of lunacy by the medical men and the indorsement of the same by the magistrate. It often occurs that the judge is not at hand after the certificate of lunacy has been signed by the physicians. It may be Saturday p. m. This makes the matter worse, for an unfortunate who should be taken directly to the asylum is left in the hands of the police who can do nothing more than confine the patient in the dreary cell of jail or lock-up, and who, in their ignorance of the needs of the case, are just as apt to handle a violent lunatic roughly as they are to abuse an excited inebriate. The effect upon the insane of such confinement and such treatment even for the short space of time between Saturday evening and Monday morning, might work years of harm and perhaps render an acute case incurable. If no police were at hand it might be excessively dangerous to the patient as well as to his friends to leave him in their care or in that of any persons who do no not know the proper manner of controlling a lunatic. Indeed much more might be said to prove the great need of a law for the protection of the insane during this transition period. The report of the committee appointed to consider this matter will not be made until the February meeting of the Councilors. But there can be but one result, and it seems singular that the importance of this necessity has not long ago been urged upon our law-makers by specialists. As it is, the proposition came from a branch of the State Society.

The next annual meeting of the Massachusetts Medical Society will be one of unusual interest, from the fact that it will be the centennial gathering of its Fellows. The exercises, of course, will have a special meaning, and there will be a decided departure from the ordinary programme. It is the intention of the president-elect to arrange a steamboat excursion among the islands of Boston harbor, or some other means of giving unusual entertainment to the members of the Society. The annual dinner will be followed by toasts and speeches which will have a most entertaining historical character. In more ways than one the remarkably harmonious and earnest spirit of the meetings and proceedings of the society for one hundred years will be prominently brought to the minds of the Fellows. This body most certainly has had a remarkable history. Probably no similar society in our country holds a higher place in the respect of the laity and its influence has ever been useful and ennobling. Many of its earlier members were men who wrought untold and enduring good in placing the profession of medicine on the elevated plane which it now occupies in the United States. The influence of their writings as well as their noble manhood was felt not only in Massachusetts, but throughout the country. Their memory is very dear to the society.

BOSTON, Oct. 16, 1880. H. O.

---

ON THE TREATMENT OF JAUNDICE WITH LARGE DOSES OF IPECAC.—Dr. Henry Cook, of Bombay, India, writes a suggestive article in the *Practitioner*, on the above subject. Jaundice he divides as usual into the hepatogenous, and hæmatogenous. The more frequent cause of the former variety is, of course, a catarrh of the gall ducts ; but, in addition there is often a form of hepatogenous jaundice, in India, at least, which comes on gradually, and is probably due to an obstruction or lesion of some kind in the minuter divisions of the hepatic duct.

Hæmatogenous jaundice, in India, is oftenest due to malarial fevers. Now, in jaundice due to any of these causes, but especially in the second form, he has found large doses of ipecac to act better than anything else. In one case he gave on the first day forty-five grains. Improvement began on the day following. On the third day he gave a dose of thirty grains, and two days later, another dose of twenty grains.

Improvement was steadily going on. In other cases two doses were sufficient to restore the normal action of the liver. Dr. Cook relates one case of hæmatogenous jaundice due to malarial fever, in which the large doses of ipecac apparently acted well.—*N. Y. Medical Record.*

